# Neuronal subset-specific *Pten*-deficient mice do not exhibit deficits in sensorimotor gating processes

**DOI:** 10.12688/f1000research.20604.3

**Published:** 2020-04-07

**Authors:** Matthew S. Binder, Suzanne O. Nolan, Joaquin N. Lugo

**Affiliations:** 1 Department of Psychology and Neuroscience, Baylor University, Waco, TX, 76798, USA; 2 Department of Pharmacology, Vanderbilt University Medical Center, Nashville, TN, 37232, USA; 3 Institute of Biomedical Studies, Baylor University, Waco, TX, 76787, USA

**Keywords:** autism, pten, macrocephaly, ASD, sensorimotor

## Abstract

**Background:** Deficits in sensorimotor gating have been reported in individuals with autism spectrum disorder (ASD), as well as in ASD murine models. However, this behavior has not been examined in the neuronal subset-specific (NS)- *Pten* knockout (KO) model of ASD. *NS-Pten* KO mice exhibit hyperactivity of the PI3K/AKT/mTOR signaling pathway which is implicated in the onset of autistic deficits. This study investigates the potential relationship between PI3K/AKT/mTOR signaling and deficits in sensorimotor gating.

**Methods:** To assess sensorimotor gating in NS- *Pten* KO mice we utilized a three-day paradigm. On day 1 (habituation) the mice were administered 80 repetitions of a 120-dB startle stimulus. On day 2, prepulse inhibition was measured with 90 trials of the startle stimulus that was paired with a smaller (2, 7, or 12 dB) prepulse stimulus. Day 3 was assessed one week later, consisting of randomized startle trials and trials with no stimulus and was used to determine the startle response.

**Results:** No significant difference between NS- *Pten* KO or wildtype (WT) mice was found for habituation ( *p* > 0.05). No significant differences were found between groups when assessing the percentage of prepulse inhibition at 2, 7, and 12 dB ( *p* > 0.05). There was also no difference in startle response between groups ( *p* > 0.05).

**Conclusion:** Our study found that the NS- *Pten* KO model does not display significant deficits in sensorimotor gating processes. The present findings help to elucidate the relationship between PI3K/AKT/mTOR hyperactivation and sensory reactivity.

## Introduction

Sensorimotor gating is the ability of a sensory stimulus to suppress a motor response ^1^. It can be measured by assessing prepulse inhibition (PPI), wherein a weak auditory stimulus inhibits a startle response that is induced by the following presentation of a loud sound ^2^. Deficits in PPI have been widely reported in various neurological conditions, including autism spectrum disorder (ASD) ^3– 5^. Similar to humans, impairments in PPI have been reported in ASD models such as *Fmr1* and *Cntnap2*-knockout (KO) mice; however, the underlying mechanism is unknown ^6, 7^. *Pten* mutant mice are another model of autism and can be used to investigate the connection between a cell signaling pathway commonly implicated in ASD, the PI3K/AKT/mTOR pathway, and specific autistic-like deficits ^8^. Specifically, neuronal subset specific (NS) *Pten* KO mice have previously been shown to exhibit deficits in repetitive behavior, sociability, and communication, however, prepulse inhibition has not been assessed in this model ^9, 10^. In the present study, we used NS- *Pten* KO mice that exhibit hyperactivation of the PI3K/AKT/mTOR pathway in the cortex, hippocampus, and cerebellum, and assess PPI in order to further elucidate the potential relationship between PI3K/AKT/mTOR signaling and deficits in sensorimotor gating ^9^.

## Methods

### Subjects

Male and female mice on a FVB based mixed background were obtained from Baylor College of Medicine and have been bred for more than 10 generations at Baylor University. Heterozygous NS- *Pten* males ( *n*=6) and females ( *n*=12) were used to breed NS- *Pten* wildtype (WT) and KO pups (RRID: MGI:3714016). The housing for the breeders consisted of two females housed with one male. Genotype was determined from toe clippings taken on postnatal day (PD) 10 (performed by Mouse Genotype, Escondido, CA, USA). On PD 21, animals were weaned and housed with mixed genotype littermates in groups of *n*=3–5 in cages (Allentown Caging PC7115HT, Allentown, PA, USA) filled with sani-chip bedding (7090 Teklad, Envigo, Somerset, NJ, USA) kept in a room on a 12-hr light/dark diurnal cycle held at 22°C. Mice had *ad libitum* access to food and water. All animals were tested at 9–10 weeks of age between the hours of 10:00 and 11:30 a.m. Only males were assessed in this study to be in accordance with the literature, as previous behavioral phenotyping studies in the NS- *Pten* adult model were done in males ^9, 10^. Furthermore, a similar study investigating sensorimotor gating in neuron-specific enolase (Nse)- *Pten* KO mice also only assessed males ^11^. Therefore, in order to make an accurate comparison and to provide similar context with past studies we did not assess females. A total of 29 male mice were assessed, 17 NS- *Pten* KO and 12 WT mice. The target sample size was determined by, and is in accordance with, the PPI literature ^11– 13^. The final sample sizes were as follows: day 1: *n*=12 WT, *n*=17 KO, day 2: *n*=12 WT, *n*=13 KO, day 3: *n*=9 WT, *n*=9 KO. A subset of n= 4 KO mice were excluded from the day 2 analysis and n = 11 mice (3 WT and 8 KO) were excluded from the day 3 analysis due to either a protocol malfunction or the death of the KO animal caused by the occurrence of spontaneous seizures. All test procedures were carried out in compliance with the NIH Guidelines for the Care and Use of Laboratory Animals and were approved by Baylor University’s Institutional Animal Care and Use Committee. Once the experiment concluded, mice were placed into a CO _2_ chamber and euthanized.

### Sensorimotor gating assessment

Sensorimotor gating was assessed via the SR-LAB system, which consists of a 15 × 14 × 18 inch isolation cabinet, a plexiglass cylinder (3.2-cm diameter) mounted on a sensor platform, and a speaker that generated white noise, as well as the stimuli (San Diego Instruments, San Diego, CA, USA). The paradigm consisted of three separate testing days: habituation, prepulse inhibition, and startle response, and was conducted as previously described ^6^.

For habituation, the animal was acclimated to the room for 30 minutes then was placed inside the cylinder for a 5-minute habituation period, which was followed by 80 startle stimuli delivered at a fixed interval of 15 seconds. The startle stimulus was a 40 ms, 120 dB noise burst, with a rise/fall time of less than 1 ms. Prepulse inhibition testing occurred 24 hours after day 1 and consisted of a 5-minute habituation phase that was followed by 20 presentations of a 40 ms, 120 dB noise burst. In the prepulse phase, mice were presented with 90 trials consisting of three prepulse intensities that were 2, 7, and 12 dB over the 68 dB background noise. The onset of the prepulse occurred 100 ms before the onset of the startle pulse. Each prepulse was 20 ms in duration and were spaced an average of 15 seconds apart (7–23 s). One week after the prepulse session, the startle response was assessed. Following the 5-minute habituation period, the mice were presented with 99 trials of 11 trial types. These included a no stimulus trial and 10 startle stimuli trials ranging from 75–120 dB at 5 dB intervals. The startle stimuli were 40 ms noise bursts. The 11 trial types were pseudorandomized, with each trial type being presented once in a block of the 11 trials. To eliminate potential confounds during testing, background sound levels were maintained at 68 dB and the experimenter was not present.

### Statistical analysis

GraphPad Prism 7 software (La Jolla, CA) or SPSS 21.0 (IBM, USA) were used to analyze the data. Repeated-measure ANOVAs were run for habituation, prepulse inhibition, and startle response. The within subject factors for habituation were the trials (1-10, 11-20, 21-30, 41-50, 51-60, 61-70, and 71-80) with genotype as the between subjects factor (wildtype and knockout). For prepulse inhibition, the within-subjects factors were the prepulse intensities (2, 7, and 12 dB), with genotype as the between subjects factor. For the startle response, the between-subjects factors were the stimulus intensities (no stimulus, startle at 75, 80, 85, 90, 95, 100, 105, 110, 115, and 120 dB), with the between subject factor of genotype. Due to the skewed data present in the startle response, a log transformation was preformed. To provide a general indicator of the overall health of each animal throughout the study, the subject’s weights were assessed with a repeated measures ANOVA that had a within subjects factor of day (weight of the subject for testing days 1, 2, and 3) and a between subjects factor of genotype. No post-hoc tests were performed. A total of *n*=4 KO mice were excluded from the day 2 analysis and *n*=11 mice (3 WT and 8 KO) were excluded from the day 3 analysis due to protocol malfunction or death as a result of the severity of the knockout. A value of *p* < 0.05 was considered significant for each statistical test.

## Results

**Figure 1.**
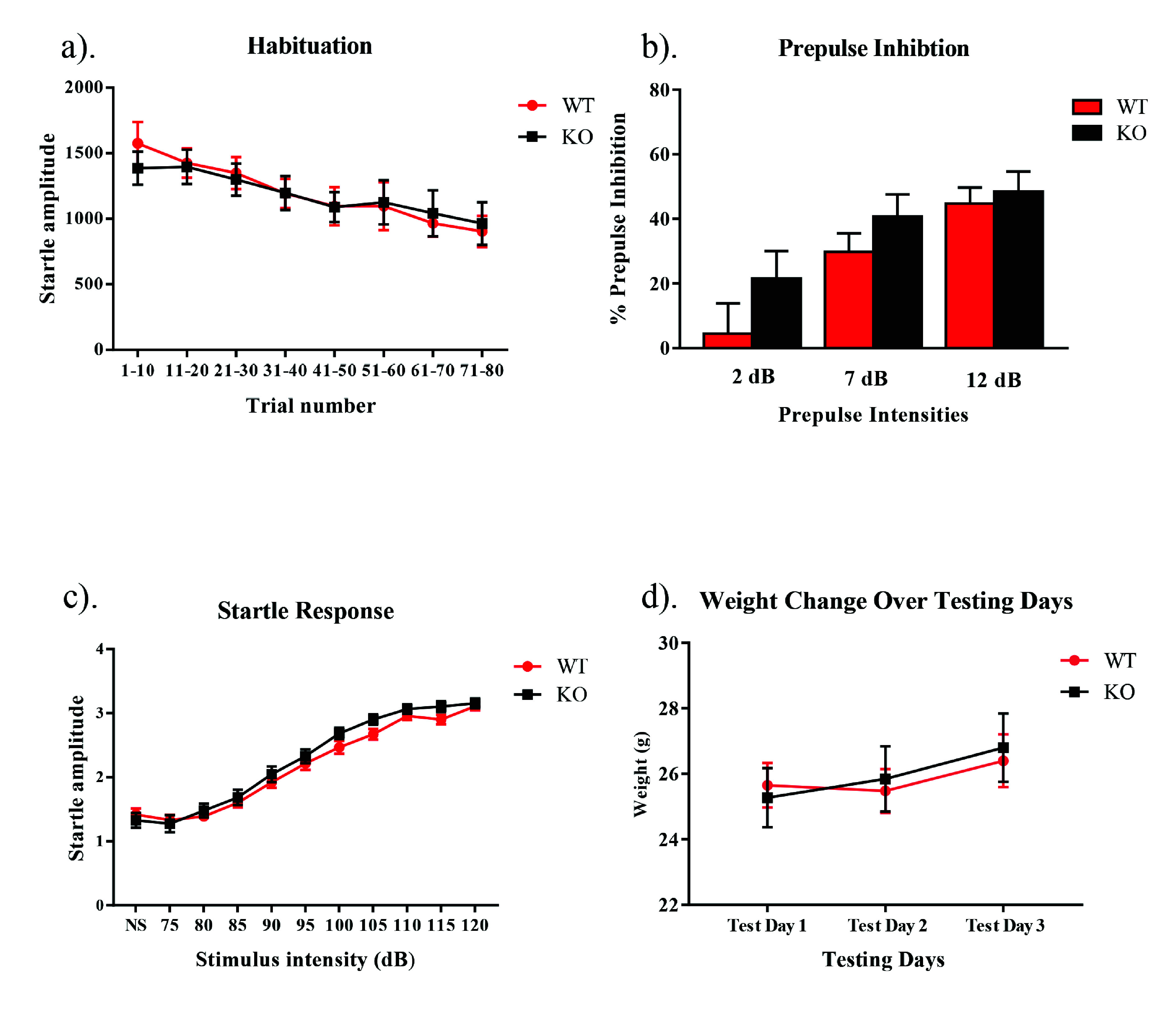
Habituation, prepulse inhibition, and startle response in NS- *Pten* KO mice. ( **a**) We found that there was no significant difference in habituation between KO and WT mice ( *p* > 0.05). ( **b**) We found no difference in the percentage of prepulse inhibition between groups following prepulses that were 2, 7, and 12 dB over the 68 dB background noise ( *p* > 0.05). ( **c**) We observed no difference in startle response between NS- *Pten* KO and WT mice ( *p* > 0.05). ( **d**) We observed no differences in weight between NS- *Pten* KO and WT mice ( *p* > 0.05). Data are presented as the mean ± standard error of the mean (SEM).

When assessing the sensorimotor gating paradigm, the main effects of genotype was not significant in the ANOVAs of the data obtained on the habituation test on day 1 ( *F*[1,27] = 0.17, *p* >0.05), the prepulse inhibition test on day 2 ( *F*[1,23] = 2.65, *p* >0.05) or the startle response test on day 3 ( *F*[1,16] = 2.10, *p* >0.05). There was also no interaction between genotype and trial for habituation ( *F*[7,189] = 0.91, *p* >0.05), genotype and prepulse intensity for prepulse inhibition ( *F*[2,46] = 0.71, *p* >0.05), or genotype and stimulus intensity for the startle response ( *F*[10,160] = .10, *p* >0.05) ( Figure 1a–c). When assessing the weight of each subject throughout the study, no main effect for the within subjects factor of day was found ( *F*[2,30] = .11, *p* > .05), nor was there a day by genotype interaction ( *F*[2,30] = .17, *p* > .05). There was also no between subjects effect of genotype ( *F*[1,15] = 1.16, *p* > .05) ( Figure 1d). Raw results for each procedure on each day for every animal are available as *Underlying data* ^13^.

## Discussion

The NS- *Pten* KO mice did not exhibit significantly different sensorimotor gating from WT mice. A previous study by Kwon *et al*. (2006) assessed neuron-specific enolase (Nse)- *Pten* KO mice in a variation of the PPI protocol and reported a decrease in percent inhibition at 4 dB but no differences at 8 or 16 dB ^11^. Our study assessed percent inhibition at 2, 7, and 12 dB, per established protocol, and found no differences at these intensities ^6^. This discrepancy in sensorimotor gating between similar models may be due to the timing of the induction of cre expression. Cre expression is induced following neuronal differentiation in Nse- *Pten* KO mice, however, in NS- *Pten* KO mice, the model we assessed, cre expression is induced prior to neuronal differentiation. Therefore, our study indicates that a premitotic expression of cre does not result in sensorimotor deficits. Additionally, in accordance with our study, no differences in prepulse inhibition have been reported in the BTBR and *Shank1* mouse models of autism ^14, 15^. Altogether, this indicates that alterations in sensory reactivity may be a less sensitive measure of an autistic-like phenotype and may also only be present in particular ASD models.

Overall, the current study found that hyperactivity of the PI3K/AKT/mTOR pathway does not result in sensorimotor gating deficits in NS- *Pten* KO mice, suggesting that the pathway may not directly affect prepulse inhibition. This conclusion is supported by a prior study that assessed PPI in a transgenic mouse model of tuberous sclerosis complex, another model of ASD and mTOR hyperactivation, which similarly reported no deficits in prepulse inhibition between WT and KO mice ^16^. Taken together, these studies indicate that despite mTOR’s contribution to an autistic-like phenotype, it does not significantly contribute to the onset of sensorimotor gating deficits in several different ASD models. Ultimately, our study contributes to the literature and suggests that the relationship between hyperactivation of the PI3K/AKT/mTOR pathway and deficits in sensory reactivity is modest.

## Data availability

### Underlying data

Figshare: Neuronal subset-specific Pten-deficient mice do not exhibit deficits in sensorimotor gating processes. https://doi.org/10.6084/m9.figshare.9885401.v2 ^13^.

This project contains the following underlying data:

- PPI Day1 Pten Raw Data 9-6.xlsx (raw data from all experiments performed for all animals; day 1).
- PPI Day 2 Pten Raw Data 9-6.xlsx (raw data from all experiments performed for all animals; day 2).
- PPI day 3 Pten Raw Data 9-6.xlsx (raw data from all experiments performed for all animals; day 3).
- Pten data weights Pten ppi weights.xlsx (raw data for weights across testing dates).

Data are available under the terms of the Creative Commons Zero “No rights reserved” data waiver (CC0 1.0 Public domain dedication).

## References

[1] Powell SB Weber M Geyer MA : Genetic models of sensorimotor gating: relevance to neuropsychiatric disorders. *Curr Top Behav Neurosci.* 2012;12:251–318. 10.1007/7854_2011_195 22367921 PMC3357439

[2] Hiroi N Nishi A : Chapter 17 - Dimensional Deconstruction and Reconstruction of CNV-Associated Neuropsychiatric Disorders. In: M.V. Pletnikov, J.L. Waddington (Eds.), *Handbook of Behavioral Neuroscience*, Elsevier.2016;23:285–302. 10.1016/B978-0-12-800981-9.00017-1

[3] Madsen GF Bilenberg N Cantio C : Increased prepulse inhibition and sensitization of the startle reflex in autistic children. *Autism Res.* 2014;7(1):94–103. 10.1002/aur.1337 24124111

[4] Mena A Ruiz-Salas JC Puentes A : Reduced Prepulse Inhibition as a Biomarker of Schizophrenia. *Front Behav Neurosci.* 2016;10:202. 10.3389/fnbeh.2016.00202 27803654 PMC5067522

[5] Takahashi H Hashimoto R Iwase M : Prepulse inhibition of startle response: recent advances in human studies of psychiatric disease. *Clin Psychopharmacol Neurosci.* 2011;9(3):102–110. 10.9758/cpn.2011.9.3.102 23429840 PMC3569113

[6] Frankland PW Wang Y Rosner B : Sensorimotor gating abnormalities in young males with fragile X syndrome and Fmr1-knockout mice. *Mol Psychiatry.* 2004;9(4):417–25. 10.1038/sj.mp.4001432 14981523

[7] Brunner D Kabitzke P He D : Comprehensive Analysis of the 16p11.2 Deletion and Null Cntnap2 Mouse Models of Autism Spectrum Disorder. *PLoS One.* 2015;10(8):e0134572. 10.1371/journal.pone.0134572 26273832 PMC4537259

[8] Song MS Salmena L Pandolfi PP : The functions and regulation of the PTEN tumour suppressor. *Nat Rev Mol Cell Biol.* 2012;13(5):283–96. 10.1038/nrm3330 22473468

[9] Lugo JN Smith GD Arbuckle EP : Deletion of PTEN produces autism-like behavioral deficits and alterations in synaptic proteins. *Front Mol Neurosci.* 2014;7:27. 10.3389/fnmol.2014.00027 24795561 PMC3997048

[10] Binder MS Jones DG Hodges SL : NS- *Pten* adult knockout mice display both quantitative and qualitative changes in urine-induced ultrasonic vocalizations. *Behav Brain Res.* 2020;378:112189. 10.1016/j.bbr.2019.112189 31586563 PMC7000110

[11] Kwon CH Luikart BW Powell CM : Pten regulates neuronal arborization and social interaction in mice. *Neuron.* 2006;50(3):377–388. 10.1016/j.neuron.2006.03.023 16675393 PMC3902853

[12] Kokash J Alderson EM Reinhard SM : Genetic reduction of MMP-9 in the Fmr1 KO mouse partially rescues prepulse inhibition of acoustic startle response. *Brain Res.* 2019;1719:24–29. 10.1016/j.brainres.2019.05.029 31128097 PMC6640842

[13] Brody SA Dulawa SC Conquet F : Assessment of a prepulse inhibition deficit in a mutant mouse lacking mGlu5 receptors. *Mol Psychiatry.* 2004;9(1):35–41. 10.1038/sj.mp.4001404 14699440

[14] Silverman JL Yang M Turner SM : Low stress reactivity and neuroendocrine factors in the BTBR T ^+^tf/J mouse model of autism. *Neuroscience.* 2010;171(4):1197–1208. 10.1016/j.neuroscience.2010.09.059 20888890 PMC2991427

[15] Silverman JL Turner SM Barkan CL : Sociability and motor functions in *Shank1* mutant mice. *Brain Res.* 2011;1380:120–137. 10.1016/j.brainres.2010.09.026 20868654 PMC3041833

[16] Chévere-Torres I Maki JM Santini E : Impaired social interactions and motor learning skills in tuberous sclerosis complex model mice expressing a dominant/negative form of tuberin. *Neurobiol Dis.* 2012;45(1):156–164. 10.1016/j.nbd.2011.07.018 21827857 PMC3225564

