# Supplementary material for: Neuronal subset-specific Pten-deficient mice do not exhibit deficits in sensorimotor gating processes
Source: F1000Res. 2025 Jan 14;8:1727. Originally published 2019 Oct 8. [Version 4] doi: 10.12688/f1000research.20604.4 (PMC11736557; doi:10.12688/f1000research.20604.4)
Supplement: Peer review history

# Reviewer report: Reviewer response for version 4

**DOI:** 10.5256/f1000research.176932.r359040

**Published:** 2025-02-05

**Authors:** George Leondaritis, Charalampos Brakatselos

**Affiliations:** 1 Mortimer B. Zuckerman Mind Brain and Behavior Institute, New York, USA; 2 Department of Pharmacology, Faculty of Medicine, and Institute of Biosciences, University Research Center of Ioannina (URCI), University of Ioannina, Ioannina, Greece

In the revised version, the discussion is much more clearer and productive. Also, the authors have highlighted potential limitations that address our concerns; these should be resolved in future studies to provide conclusive evidence of the PPI endophenotype in PTEN/mTOR-related ASD mouse models. As such, the current paper constitutes an important contribution towards this goal.

Is the work clearly and accurately presented and does it cite the current literature?

Yes

If applicable, is the statistical analysis and its interpretation appropriate?

Yes

Are all the source data underlying the results available to ensure full reproducibility?

Yes

Is the study design appropriate and is the work technically sound?

Partly

Are the conclusions drawn adequately supported by the results?

Partly

Are sufficient details of methods and analysis provided to allow replication by others?

Yes

Reviewer Expertise:

George Leondaritis (PTEN/mTOR signaling, neurodevelopmental disease, neuronal differentiation)Charalampos Brakatselos(mental disease modeling, neuropsychopharmacology, behavioral neuroscience)

We confirm that we have read this submission and believe that we have an appropriate level of expertise to confirm that it is of an acceptable scientific standard.

# Reviewer report: Reviewer response for version 4

**DOI:** 10.5256/f1000research.176932.r359041

**Published:** 2025-01-30

**Authors:** Maarten van den Buuse

**Affiliations:** 1 School of Psychology and Public Health, La Trobe University, Melbourne, Vic, Australia

Thank you for the opportunity to review this manuscript again. I believe the authors have responded well to my comments and have made appropriate changes to their paper. I have no further comments.

Is the work clearly and accurately presented and does it cite the current literature?

Partly

If applicable, is the statistical analysis and its interpretation appropriate?

No

Are all the source data underlying the results available to ensure full reproducibility?

No source data required

Is the study design appropriate and is the work technically sound?

Yes

Are the conclusions drawn adequately supported by the results?

Partly

Are sufficient details of methods and analysis provided to allow replication by others?

Partly

Reviewer Expertise:

Prepulse inhibition, animal models of psychiatric disease.

I confirm that I have read this submission and believe that I have an appropriate level of expertise to confirm that it is of an acceptable scientific standard.

# Reviewer report: Reviewer response for version 3

**DOI:** 10.5256/f1000research.25489.r343972

**Published:** 2025-01-02

**Authors:** George Leondaritis, Charalampos Brakatselos

**Affiliations:** 1 Department of Pharmacology, Faculty of Medicine, and Institute of Biosciences, University Research Center of Ioannina (URCI), University of Ioannina, Ioannina, Greece; 2 Mortimer B. Zuckerman Mind Brain and Behavior Institute, New York, USA

The manuscript by Binder et al addresses a very interesting question in the field of Pten and mTOR, two signaling molecules that have provided a strong connection to ASD in humans. In essence, the authors demonstrate that a specific Pten KO mouse model, to which they refer to as “neuronal subset specific” or NS-Pten KO, does not exhibit significantly different sensorimotor gating compared to WT mice, using a Prepulse Inhibition (PPI) protocol. The authors discuss their findings with respect to other studies assessing PPI deficits in several ASD mouse models that are deficient in signaling proteins of the mTOR pathway, including a different Pten KO mouse model that was previously shown to display PPI deficits ^1^.

 The study thus addresses the inconsistencies in findings of sensorimotor gating deficiencies in ASD mouse models. PPI is an important clinical and preclinical endophenotype, mainly associated with schizophrenia, but also frequently impaired in other neurological disorders. The literature associating PPI deficits with ASD remains elusive, so any additions could be of great value.

 Although the methodological implementation for the specific protocol that was followed seems reliable and the data analysis and statistics are transparent, we have two concerns that, if addressed properly, they would significantly strengthen the major points of the manuscript.

 1) Our first concern relates to the PPI protocol that was implemented. The authors selected a protocol that is implemented in a 3-day regiment for every mouse, while the actual analysis of the PPI index takes place only during the 2 ^nd^ day. Startle amplitude and PPI testing are considered a relatively stressful experience for the mice, while PPI outcomes are known to be disturbed by stressful experiences. Is it possible that a potential PPI deficit phenotype could have been masked by the stressful experience of the sustained exposure to the PPI chamber (especially given that Day 1 includes 80 startle representations, while there is no effect on startle response amplitude or habituation)? Could minor differences concerning acclimation to the experimental space (i.e., at least 5 days before testing), or habituation to the room (i.e., 45-60 min rather than 30min) also play a role in masking a subtle but still significant deficit? It is important to consider that the +2 and +7 db (above the background) prepulse intensities are displaying quite considerable effect size changes between WT and KO mice.

 Another consideration relates to the nature of the pulse tone. It would be helpful to clarify whether the Prepulse and Pulse tones were specific frequencies or white noise; while this is clear for the background noise, it is not for the tones. This aspect is important as auditory/perceptual deficits could be expected in some mouse lines associated with neuropsychiatric disorders. In the case of auditory deficits, white noise is preferable as the deficit could be specific for a subset of frequencies of the hearing range.

 Perhaps the authors could discuss these issues and/or highlight appropriate limitations of their study along the above directions.

 2) The second concern relates to the description of the NS Pten KO model. The authors compare the absence of PPI deficits in this Pten model with the presence of PPI deficits observed in another Pten model, namely the NSE-Pten KO mice ^1^. The authors should elaborate a bit more on the differences of the two Pten models, as they do not only differ in the timing of Cre expression relative to onset of neuronal differentiation. The NS-Pten KO mice that the authors studied is apparently the GFAP-Cre Pten KO model characterized previously as a Lerhmitte-Duclos syndrome model ^2,3^. This information, as well as the differences in the extent and cell-type dependency of PTEN deletion in different brain regions of the GFAP- and NSE-Cre KO models, remain unclear throughout the manuscript. The authors should elaborate here for the sake of readers that are not PTEN-specialists; this will certainly assist the comprehension of the study’s significance. For example, and relative to the authors’ comment in the 2 ^nd^ paragraph of the discussion section, is the level of PI3K /AKT/mTOR pathway hyperactivation in the two PTEN models similar ?  Another distinguishing feature of the two models may relate to the seizure phenotype of the GFAP-Cre PTEN mice. These mice suffer from seizures at an early age ^2,3^, with tonic-clonic seizures appearing between wks 7 and 10 and the authors do report that some of the KO mice died due to spontaneous seizures. On the other hand, the NSE-Cre Pten KO mice show relatively low incidences and short duration of seizures ^1^. Is it thus possible that the absence of a PPI deficit could relate to the seizure phenotype of the NS-Pten (aka GFAP-Cre PTEN KO) mice?

 Conclusively, we feel that the authors should elaborate a bit more on the differences of the two Pten mouse models they get to compare. It would seem likely that differences in Pten/mTOR dysregulation at either specific developmental stages or specific neuron types, or even associated co-morbitities, might be a determining factor for detecting an ASD-related PPI endophenotype.

Is the work clearly and accurately presented and does it cite the current literature?

Yes

If applicable, is the statistical analysis and its interpretation appropriate?

Yes

Are all the source data underlying the results available to ensure full reproducibility?

Yes

Is the study design appropriate and is the work technically sound?

Partly

Are the conclusions drawn adequately supported by the results?

Partly

Are sufficient details of methods and analysis provided to allow replication by others?

Yes

Reviewer Expertise:

George Leondaritis (PTEN/mTOR signaling, neurodevelopmental disease, neuronal differentiation)Charalampos Brakatselos(mental disease modeling, neuropsychopharmacology, behavioral neuroscience)

We confirm that we have read this submission and believe that we have an appropriate level of expertise to confirm that it is of an acceptable scientific standard, however we have significant reservations, as outlined above.

## References

[1] : Pten regulates neuronal arborization and social interaction in mice. *Neuron* .2006;50(3) : 10.1016/j.neuron.2006.03.023 377-88 10.1016/j.neuron.2006.03.023 16675393 PMC3902853

[2] : Deletion of Pten in mouse brain causes seizures, ataxia and defects in soma size resembling Lhermitte-Duclos disease. *Nat Genet* .2001;29(4) : 10.1038/ng782 396-403 10.1038/ng782 11726926

[3] : Pten regulates neuronal soma size: a mouse model of Lhermitte-Duclos disease. *Nat Genet* .2001;29(4) : 10.1038/ng781 404-11 10.1038/ng781 11726927

# Response

**Published:** 2025-01-10

**Authors:** Joaquin Lugo

**Affiliations:** Baylor University, USA

Response1: Thank you for your comments. We have added a paragraph to the conclusion that discusses specific limitations of our study.   We have also clarified that white noise bursts were used in the methods section of the paper

 Response2: You raise excellent points. We have significantly expanded the discussion section to more precisely detail differences between our model and the comparison studies. We have also clarified that the level of mTOR activation is similar across the models. Lastly, we have discussed the seizure phenotype in both models in more depth and acknowledged that seizures may have had a possible effect on sensorimotor gating behavior.

# Reviewer report: Reviewer response for version 3

**DOI:** 10.5256/f1000research.25489.r62086

**Published:** 2020-04-14

**Authors:** Benjamin K. Yee

**Affiliations:** 1 Department of Rehabilitation Sciences, Hong Kong Polytechnic University, Hong Kong, China

I have read through the replies by the authors, and I think they have provided sufficient efforts to address my previous concerns. 

 The statistically null effect of the mutation on PPI is clear but it still does not constitute direct support for a lack of effect (given the intrinsic nature of hypothesis testing). 

 Nonetheless, the outcome does represent a contrast to some existing reports and would be instrumental in illustrate the fragility of the published finding. To this end, the paper does represents a contribution.

Is the work clearly and accurately presented and does it cite the current literature?

Partly

If applicable, is the statistical analysis and its interpretation appropriate?

Partly

Are all the source data underlying the results available to ensure full reproducibility?

Partly

Is the study design appropriate and is the work technically sound?

Partly

Are the conclusions drawn adequately supported by the results?

Partly

Are sufficient details of methods and analysis provided to allow replication by others?

No

Reviewer Expertise:

NA

I confirm that I have read this submission and believe that I have an appropriate level of expertise to confirm that it is of an acceptable scientific standard.

# Reviewer report: Reviewer response for version 2

**DOI:** 10.5256/f1000research.24639.r59885

**Published:** 2020-02-12

**Authors:** Benjamin K. Yee

**Affiliations:** 1 Department of Rehabilitation Sciences, Hong Kong Polytechnic University, Hong Kong, China

While the interpretation that neuron-specific deletion of (NS)-Pten did not substantially alter the acoustic reflex startle (ASR) and PPI of ASR largely agrees with the impression of the data presented, the reporting of the data is not sufficiently stringent. Some responses to the previous comments are inadequate.

 Please rephrase the sentence: “When assessing the sensorimotor gating paradigm, there were no main effects of genotype for habituation (F[1,27] = 0.17, p >0.05), prepulse inhibition (F[1,23] = 2.65, p >0.05) or startle threshold (F[1,16] = 2.33, p >0.05).”

 This sentence is awkward because of the word “for”, I think. It simply intends to indicate that the main effect of genotype is not significant in the ANOVAs of the data obtained on the habituation test on day 1, the prepulse inhibition test on day 2, and the startle reactivity profile on day 3. My comment before focuses on the fact that the main effect of genotype cannot inform on the habituation of startle response.

 Legends of Figure 1: Description of (a) and (c) have been mixed up. Either correct the legends or the placement/labels of the two plots.

 The reasons behind the drop of animals from Day 1 to Day 3 should be provided in the text, although it is available from the links to the raw data files.

 Mouse 2355 was reportedly dead on Day 2 but provided body weight on Day 3 (see data file - pten ppi weights.xlsx in the underlying data).

 It appears that the total number of animals included in the weights analysis across days was 17. It was specified that Day 3 test comprised 9 + 9 = 18 mice. One mouse’s data was missing. I suspect the missing mouse was the one reported dead on Day 2 but not dead on Day 3.

 The data distribution of the data set for “threshold analysis” was highly skewed. It may be appropriate to log transform the data set, which incidentally strengthen the impression that the two genotypes were closed matched in their reactivity profile (as a function of stimulus intensity).

 The response to the critique concerning the lack of any attempt to index (indeed, or to define) “threshold” is inadequate. The group by intensity interaction in the ANOVA evaluate the extent to which the two reactivity curves (as function of stimulus intensity) are parallel. The main effect of genotype evaluated the average “height” of the reactivity curve between groups. While it may be true that the genotype and genotype by stimulus intensity did not reach statistical significance. Neither of them directly addresses “threshold”. One may admit that if the two lines are indeed identical and thus no difference in threshold would be possible (no matter how one may define it), then neither the main effect of genotype nor the interaction would achieve statistical significance. The reverse argument does not necessarily stand.

 The authors may consult this article by Hince *et al.* ^1^ for curve fitting methods to obtain proxies for a startle threshold value (e.g., stimulus intensity that yield 50% of the maximum reaction generated by the most intense stimulus) for individual animals.

 My previous comment was not clear because I had examined the data set in question trial-by-trial, rather than just block-by-block in the reported ANOVA of data on Day 1. Below is the plot that clearly shows that the startle reaction obtained in trial 1 was notably weaker than the rest. This anomaly warrants an explanation. First, this is highly unusual. Typically we expect the highest response in the first few trials. Second, Day 1 was designed to examine habituation, yet it produced a strong sensitization effect from trial 1 to trial 2. Some checking on the SR-LAB programme or derivation of raw data may be warranted, even though it is not likely that a systemic error in, and if limited to, a single trial could substantially change the statistical outcomes.

Plot

 The final concluding sentence warrants revision. it reads “…our study is in support of the literature and helps to further elucidate the relationship between hyperactivation of the PI3K/AKT/mTOR pathway and deficits in sensory reactivity”.

 However, the present null results, if anything, are not supportive of a functional link between hyperactivation of the PI3K/AKT/mTOR pathway (as a result of the genetic knockout) and any deficits in sensory reactivity as exemplified by the acoustic startle reflex and its modulation in the form of PPI.

Is the work clearly and accurately presented and does it cite the current literature?

Partly

If applicable, is the statistical analysis and its interpretation appropriate?

Partly

Are all the source data underlying the results available to ensure full reproducibility?

Partly

Is the study design appropriate and is the work technically sound?

Partly

Are the conclusions drawn adequately supported by the results?

Partly

Are sufficient details of methods and analysis provided to allow replication by others?

No

Reviewer Expertise:

Prepulse inhibition, Behavioural phenotyping of mutant mice, animal models of schizophrenia

I confirm that I have read this submission and believe that I have an appropriate level of expertise to confirm that it is of an acceptable scientific standard, however I have significant reservations, as outlined above.

## References

[1] : Differences in prepulse inhibition (PPI) between Wistar and Sprague-Dawley rats clarified by a new method of PPI standardization. *Behav Neurosci* .2005;119(1) : 10.1037/0735-7044.119.1.66 66-77 10.1037/0735-7044.119.1.66 15727513

# Response

**Published:** 2020-03-30

**Authors:** Joaquin Lugo

**Affiliations:** Baylor University, USA

Dear Reviewer,

Please see our rebuttal for your comments. We appreciate your input and believe the paper has been improved as a result of your input. Thank you.

1. Thank you for your comment, the sentence has been modified to state: “When assessing the sensorimotor gating paradigm, the main effect of genotype was not significant in the ANOVAs of the data obtained on the  habituation test on day 1 ( *F*[1,27] = 0.17, *p* >0.05), the prepulse inhibition test on day 2 ( *F*[1,23] = 2.65, *p* >0.05) or the startle response test on day 3 ( *F*[1,16] = 2.10, *p* >0.05)” to clarify our results.

2. The figure legend has been changed to reflect that the habitation data is depicted in figure 1a and the startle response data is depicted in figure 1c.

3. Thank you for your suggestion the following sentence has been added to the subjects section of the methods. “A subset of *n*= 4 KO mice were excluded from the day 2 analysis and *n* = 11 mice (3 WT and 8 KO) were excluded from the day 3 analysis due to either a protocol malfunction or the death of the KO animal caused by the occurrence of spontaneous seizures.”

4. Per your insight, mouse 2355’s weight for day 2 has been added to the excel weight spreadsheet, as the animal survived throughout testing.  

5. The excel page has been updated to include the missing weight data to reflect the sample size used in analysis.

6. The data was transformed using a log transformation per your insight, and analysis for the startle analysis were rerun. No differences were detected, the results and methods section have been updated to reflect this transformation. 

7. Thank you for clarifying your previous comment. You raise a terrific point; we should not have referred to graph as startle threshold as it depicts the average startle response at each decibel level which does not in and of itself constitute a threshold. Therefore, the graph title has been changed to: “Average Startle Response.” All other references to this data as startle threshold have been replaced with “Startle Response”. Importantly, the analysis we ran for startle amplitude is in line with other papers in the field such as Frankland et al., (2004), Brody & Geyer (2004) etc. which was done to maximize our paper’s points of comparison.

Frankland, P., Wang, Y., Rosner, B. *et al.* Sensorimotor gating abnormalities in young males with fragile X syndrome and *Fmr1*-knockout mice. *Mol Psychiatry* **9, **417–425 (2004). https://doi.org/10.1038/sj.mp.4001432

Brody, S.A., Geyer, M.A. Interactions of the mGluR5 gene with breeding and maternal factors on startle and prepulse inhibition in mice. *neurotox res* **6, **79–90 (2004). https://doi.org/10.1007/BF03033300

8. Please see preceding response.

9. To ensure clarity, is the graph in question Day 1 Habituation (Figure 1a) or is it the Day 3 Startle Response (Figure 1c)? The Day 1 Habituation graph does not appear to conform to the pattern you are describing, as the first trial (1-10) exhibits the highest startle amplitude of approximately 1500 for WT and KO mice whereas the next trial (11-20) are below 1500, making the first trial the one with the highest startle amplitude for Day 1 Habituation. Moreover, the pattern of habituation for the remainder  of the trial conforms to an expected trend (Schmid et al., 2011). Furthermore, all SR-LAB protocols and data were rechecked, re-exported, and re-analyzed and the exact same figure was generated. Therefore, since the protocol is functioning as expected and the rest of the data points display the anticipated pattern, and the pattern we observed has been previously reported, it indicates that there was no systemic error present.         

Meanwhile, the Day 3 Startle Response has a slightly higher no stimulus startle amplitude than the 75 dB stimulus, which may be what you are referring to. Importantly, Frankland et al., (2004) also reported a higher startle amplitude for the no stimulus trial and a lower startle amplitude for the second trial, similar to our findings, reinforcing our observations. Additionally, it is significant to note that the difference between trials 1 and 2 is slight and that the rest of the trials conformed to the expected curve (Gould et al., 2004). Once again, all SR-LAB protocols and data were rechecked, re-exported, and re-analyzed and the exact same figure was generated. Therefore, since the protocol is functioning as expected and the rest of the data points display the anticipated pattern, and the pattern we observed has been previously reported, it again indicates that there was no systemic error present. Additionally, when the 75 dB stimulus intensity trial is removed from analysis no significant effects were again found, therefore, the lack of significance reported for Day 3 Startle response was not dependent upon that one trial. Ultimately, we elected to keep the 75 dB trial in the results section of the paper and in the figure in order to be transparent with our data and to judicially report all findings, as the observed data points in question were not due to protocol malfunction or other confounds and did not bias statistical analysis.  

Schmid, S., Azzopardi, E., De Jaeger, X., Prado, M.A.M. and Prado, V.F. (2011), VAChT knock‐down mice show normal prepulse inhibition but disrupted long‐term habituation. Genes, Brain and Behavior, 10: 457-464. doi: 10.1111/j.1601-183X.2011.00686.x

Frankland, P., Wang, Y., Rosner, B. *et al.* Sensorimotor gating abnormalities in young males with fragile X syndrome and *Fmr1*-knockout mice. *Mol Psychiatry* **9, **417–425 (2004). https://doi.org/10.1038/sj.mp.4001432

Gould TJ, Bizily SP, Tokarczyk J, et al. Sensorimotor gating deficits in transgenic mice expressing a constitutively active form of Gs alpha. *Neuropsychopharmacology*. 2004;29(3):494–501. doi:10.1038/sj.npp.1300309

10. Thank you for your suggestion, the final sentence has been changed to be more precise and states: “Ultimately, our study contributes to the literature and suggests that the relationship between hyperactivation of the PI3K/AKT/mTOR pathway and deficits in sensory reactivity is modest.”

# Reviewer report: Reviewer response for version 1

**DOI:** 10.5256/f1000research.22655.r54867

**Published:** 2019-11-08

**Authors:** Maarten van den Buuse

**Affiliations:** 1 School of Psychology and Public Health, La Trobe University, Melbourne, Vic, Australia

This Brief Report shows that a specific Pten-deficient mouse model shows no change in prepulse inhibition compared to wildtype controls. This is discussed in the context of literature about changes in PPI in humans with autism and in autism animal models. Albeit negative, this report could then be of value as an additional component of this literature. Unfortunately there are some problems with the study and the way it is presented.

 Abstract, background:

- "has been rarely examined” - does this mean it has been examined once before? What was the result?

Introduction:

- It would be helpful if the neuronal subset-specific Pten KO mouse was described in more detail. What do we know about behavioural changes in this mouse model. Importantly, were there deficits in social behaviour? If this has not been published yet, it would be good to add some of those additional behavioural tests here.

 

 Methods, subjects:

- Why were only males included in the study?

Methods, sensorimotor gating:

- As far as I know, the SR-LAB system has only one speaker and there is no “high-frequency” speaker (what is that anyway) to produce the stimuli.

Methods, sensorimotor gating:

- It would be more clear if the prepulses were described as level over background, i.e. PP2, PP7 and PP12.

Methods, sensorimotor gating:

- What was the interval between the onset of the prepulse and the onset of the startle pulse (SOA)? What was the interval between various trials in the PPI protocol (ITI)? Is it, like the figure legend suggests, always 15 seconds? This would be unusual because the ITI is variable in most of the PPI literature.

Results:

- Details of the statistical analysis are missing. In the Statistical Analysis section in the Methods, or in the Results section, it has to be explained what the between-group and within-group factors are and main effects and importantly interactions between those factors have to be detailed. For example, for PPI, was there a prepulse intensity x genotype interaction?

Discussion:

- Again, it would be more clear if the prepulses were described as level over background, i.e. PP2, PP7 and PP12. This would allow better comparison with previous studies.

 

 Figure 1:

- Along the horizontal axis do not use labels at an angle.

Figure 1, legend:

- A lot of technical detail here should be included in the Methods section, not in a figure legend. There is also no need to constantly repeat rise-fall times. This can be mentioned once in the Methods as a feature of all stimuli.

Is the work clearly and accurately presented and does it cite the current literature?

Partly

If applicable, is the statistical analysis and its interpretation appropriate?

No

Are all the source data underlying the results available to ensure full reproducibility?

No source data required

Is the study design appropriate and is the work technically sound?

Yes

Are the conclusions drawn adequately supported by the results?

Partly

Are sufficient details of methods and analysis provided to allow replication by others?

Partly

Reviewer Expertise:

Prepulse inhibition, animal models of psychiatric disease.

I confirm that I have read this submission and believe that I have an appropriate level of expertise to confirm that it is of an acceptable scientific standard, however I have significant reservations, as outlined above.

# Response

**Published:** 2020-02-04

**Authors:** Joaquin Lugo

**Affiliations:** Baylor University, USA

Reviewer 2. The original comments are first followed by a bullet point with our response.

Abstract, background:

"has been rarely examined” - does this mean it has been examined once before? What was the result?

- Thank you for your comment. The sentence has been modified to clarify that no other study has assessed prepulse inhibition in neuronal subset specific KO mice.

 

Introduction:

It would be helpful if the neuronal subset-specific Pten KO mouse was described in more detail. What do we know about behavioural changes in this mouse model. Importantly, were there deficits in social behaviour? If this has not been published yet, it would be good to add some of those additional behavioural tests here.

- Per your insight, a sentence has been added into the introduction that details the established behavioral phenotype of NS- *Pten* KO mice, clarifying that this model presents with deficits in repetitive behavior, sociability, and communication.

 

Methods, subjects:

Why were only males included in the study?

- Previous studies investigating the adult phenotype in the NS- *Pten* model only assessed males (see citations 9 and 10 in the document). Moreover, a similar study that assessed PPI using the Nse- *Pten* mouse also only investigated males (citation 11). In order to best align our study with other pertinent studies, and to provide similar points of comparison, we also only assessed male mice, acting in accordance with the literature. 

 

Methods, sensorimotor gating:

As far as I know, the SR-LAB system has only one speaker and there is no “high-frequency” speaker (what is that anyway) to produce the stimuli.

- It has been clarified in the methods that 1 speaker was used to generate the white noise and startle stimuli.

               

Methods, sensorimotor gating:

It would be more clear if the prepulses were described as level over background, i.e. PP2, PP7 and PP12.

-  Thank you for your suggestion, the methods have been changed to discuss the prepulses used as being relative to the background noise. 

 

Methods, sensorimotor gating:

What was the interval between the onset of the prepulse and the onset of the startle pulse (SOA)? What was the interval between various trials in the PPI protocol (ITI)? Is it, like the figure legend suggests, always 15 seconds? This would be unusual because the ITI is variable in most of the PPI literature.

- The methods section has been modified to state that the SOA was 100 ms and to clarify that the ITI in the PPI procedure was an average of 15 seconds with individual trials ranging from 7-23 seconds. 

 

Results:

Details of the statistical analysis are missing. In the Statistical Analysis section in the Methods, or in the Results section, it has to be explained what the between-group and within-group factors are and main effects and importantly interactions between those factors have to be detailed. For example, for PPI, was there a prepulse intensity x genotype interaction?

- Per your insight, the statistical analysis section now clarifies what the between subjects and within subjects’ factors are for each test day. Additionally, the results section has been amended to make it clearer that there were no main effects or interactions present for any test day.

 

Discussion:

Again, it would be more clear if the prepulses were described as level over background, i.e. PP2, PP7 and PP12. This would allow better comparison with previous studies.

-  The discussion section now refers to the prepulses in terms of their increase over the background level (ppi 2,7, and 12 dB) in order to make clearer comparisons to other studies. 

 

Figure 1:

Along the horizontal axis do not use labels at an angle.

-  The labels on the x axis for all figures are now horizontal.

 

Figure 1, legend:

A lot of technical detail here should be included in the Methods section, not in a figure legend. There is also no need to constantly repeat rise-fall times. This can be mentioned once in the Methods as a feature of all stimuli.

- The detail concerning each testing day has been moved to the methods section. Also, the rise fall times are now only mentioned once.

# Reviewer report: Reviewer response for version 1

**DOI:** 10.5256/f1000research.22655.r55104

**Published:** 2019-10-16

**Authors:** Benjamin K. Yee

**Affiliations:** 1 Department of Rehabilitation Sciences, Hong Kong Polytechnic University, Hong Kong, China

Whether sensorimotor gating, as evaluated by the prepulse inhibition (PPI) of the acoustic startle reflex paradigm, is attenuated or exaggerated in ASD is still controversial. The present study attempted to investigate this using a mutant mouse model. Specifically, neuronal deletion of Pten in the mouse is expected to result in P13K/AKT/mTOR hyperactivity implicated in ASD onset. The study may potentially clarify whether this genetic manipulation would be sufficient to modify PPI expression. No difference between mutants and wild type (WT) mice was reported. Indeed, the magnitude, habituation and threshold of the startle response as such were reported to be highly comparable between genotypes. The null results led the authors to conclude that the contribution of the mTOR pathway to ASD-related PPI deficits is limited. Closer examination of the methods and data reveal significant concerns that undermine confidence in the reliability and robustness of the reported findings.

  

1. No attempt was made to examine sex difference, while it is highly relevant to ASD.
2. Methodology details were not sufficient. Essential test parameters such as ITI and SOA in prepulse-pulse trials were not reported. Wide of response window was not reported, although it could be discerned from the raw data file.
3. Apparently, 8 (out of 17) mutant mice died from Experiment 1 to Experiment 3. This led one to suspect that the mutant mice had serious and widespread physiological defects, which could undermine any meaningful comparison. One would like to see body weights reported at least. Were the mutants significantly lighter?
4. Statistical results are poorly reported. Only “main effects” (supposedly the genotype effects) were considered. Statistics towards ascertaining the presence of startle habituation (e.g., Trials or blocks of 10 trials effects), and prepulse inhibition (the effect of prepulse intensities) etc. are not provided. To report that “no main effects were found for habituation F(1,27)=…” is inappropriate, because the comparison of habituation between genotypes could only be meaningfully evaluated by reference to the Genotype x Blocks of 10 trials interaction. Reporting the main effect of Genotype does not allow an effective assessment of the habituation profile, merely the overall magnitude of startles.
5. The plot shown in Figure 1a cannot be reproduced from the raw data provided.
6. It is also observed that the first trial of Day 1 data were all very low (in all mice). This is highly unusual and may indicate a protocol failure, or misalignment of data.
7. Examination of Day 2 data for PPI assessment also reveals another anomaly. At least 4 mice (ID: 2081, 2084, 2085, 2072) exhibited very weak startle values (well under 100) in all “120startle” trials – substantially lower than the startle magnitude obtained on the previous startle habituation test. The change is massive and inexplicable. The problem may be more extensive and include other mice. The authors should exercise due diligence in examining their data before analysis.
8. In their discussion of Kwon et al.’s (2006) reported findings of a PPI deficit (Nse)-Pten KO mice, the authors mistook the prepulses at 4dBm 8dB and 16dB as the actual magnitude of the prespulses used by Kwon et al. In fact, these refer to prepulse of intensity at 4, 6 and 18 decibels units above background. The use of 70, 75 and 80dB prepulses here were presented against a background noise level of 68dB, and thus effectively be +2, +7 and +12 decibel units above background. Hence, it is incorrect to conclude (by comparison between the present study and Kwon et al.) that “there may only be changes in percent inhibition in Pten mutant mice when the prepulse in comparatively quiet”.  If anything, the +2 (or 70 dB) condition here was even lower than the weakest prepulse used by Kwon et al.
9. The authors evaluated the startle reactivity curve as a function of increasing pulse intensity – as a means to examine the “startle threshold”. Yet no attempt was made to measure individual startle threshold for comparison between genotypes. Otherwise, it is misleading to conclude that threshold did not differ when only the group’s average profile was presented.

 

 Hence, although the available data tend to support the overall lack of an effect of the gene KO on PPI, the methods, presentation, data analysis are clearly inadequate.

Is the work clearly and accurately presented and does it cite the current literature?

Partly

If applicable, is the statistical analysis and its interpretation appropriate?

Partly

Are all the source data underlying the results available to ensure full reproducibility?

Partly

Is the study design appropriate and is the work technically sound?

Partly

Are the conclusions drawn adequately supported by the results?

Partly

Are sufficient details of methods and analysis provided to allow replication by others?

No

Reviewer Expertise:

Prepulse inhibition, Behavioural phenotyping of mutant mice, animal models of schizophrenia

I confirm that I have read this submission and believe that I have an appropriate level of expertise to state that I do not consider it to be of an acceptable scientific standard, for reasons outlined above.

# Response

**Published:** 2020-02-04

**Authors:** Joaquin Lugo

**Affiliations:** Baylor University, USA

Reviewer comments are first followed by a bullet point of our response.

No attempt was made to examine sex difference, while it is highly relevant to ASD.

- Thank you for your comment. Previous studies investigating the adult phenotype in the NS- *Pten* model only assessed males (see citations 9 and 10 in the document). Moreover, a similar study that assessed PPI using the Nse- *Pten* mouse also only investigated males (citation 11). In order to best align our study with other pertinent studies, and to provide similar points of comparison, we also only assessed male mice, acting in accordance with the literature. 

 

Methodology details were not sufficient. Essential test parameters such as ITI and SOA in prepulse-pulse trials were not reported. Wide of response window was not reported, although it could be discerned from the raw data file.

-  The methods section has been modified to state that the SOA was 100 ms and to clarify that the ITI in the PPI procedure was an average of 15 seconds with individual trials ranging from 7-23 seconds. 

 

Apparently, 8 (out of 17) mutant mice died from Experiment 1 to Experiment 3. This led one to suspect that the mutant mice had serious and widespread physiological defects, which could undermine any meaningful comparison. One would like to see body weights reported at least. Were the mutants significantly lighter?

- Per your suggestion, the weight data for WT and KO mice across each testing timepoint were analyzed. No differences in weight were found between WT and KO mice at any test point. NS- *Pten* KO mice do present with spontaneous seizures that can result in death, however, the KO mice that did not die prematurely did not display a significantly different weight from the controls, indicating that their constitution was sufficient to reliably assess the effects of PPI. Furthermore, our timepoints of testing are in accordance with the literature, making our comparison with other studies valid. Lastly, a graph of the weight data comparing WT to control mice per each testing timepoint has been created and has been uploaded.   

 

Statistical results are poorly reported. Only “main effects” (supposedly the genotype effects) were considered. Statistics towards ascertaining the presence of startle habituation (e.g., Trials or blocks of 10 trials effects), and prepulse inhibition (the effect of prepulse intensities) etc. are not provided. To report that “no main effects were found for habituation F(1,27)=…” is inappropriate, because the comparison of habituation between genotypes could only be meaningfully evaluated by reference to the Genotype x Blocks of 10 trials interaction. Reporting the main effect of Genotype does not allow an effective assessment of the habituation profile, merely the overall magnitude of startles.

- Thank you for your input, the results section has been updated to better specify the statistical tests run and the corresponding results. Additionally, the overall statistical design of the study has been added to the statistical analysis section in the methods. Regarding the statistical measure used, we agree with you that a main effect by itself is not sufficient to best assess the data, that is why we also included the statistics for the interactions of each test. For habituation, no interactions were found, indicating that the stated results are an effective assessment of the habituation profile and that our statistics were not improper. In light of your comment, the results section has been reworded in order to better highlight this and to clarify any ambiguity. 

 

The plot shown in Figure 1a cannot be reproduced from the raw data provided. 

- The plot shown in figure 1 a was created by taking the data in the T-AB columns in the excel document for the habituation day then pasting them into a grouped data file in Graphpad. The x axis for the grouped file was the trials 1-10, 11-20, etc and group A was the WT whereas group B was the KO. All data analyzed and graphed came from the corresponding excel documents. 

 

It is also observed that the first trial of Day 1 data were all very low (in all mice). This is highly unusual and may indicate a protocol failure, or misalignment of data.

- We do not understand this comment. The first trial in day 1 shows the largest startle response. Please clarify your comment.

 

Examination of Day 2 data for PPI assessment also reveals another anomaly. At least 4 mice (ID: 2081, 2084, 2085, 2072) exhibited very weak startle values (well under 100) in all “120startle” trials – substantially lower than the startle magnitude obtained on the previous startle habituation test. The change is massive and inexplicable. The problem may be more extensive and include other mice. The authors should exercise due diligence in examining their data before analysis.

-  We ran additional analysis to examine this, specifically, the mice in question were removed from analysis and the analysis was rerun excluding them, no difference between genotype was found ( *F*(1,20) = .17, *p* = .69). Therefore, the results and conclusions in the paper remain consistent. Additionally, the protocol run for those mice was in compliance with all of the other trials and no oddities were documented, indicating that the lower values may be an artifact of the mouse that was being run. Due to this, and to avoid undue manipulation within the groups, all of the mice were included.

 

In their discussion of Kwon et al.’s (2006) reported findings of a PPI deficit (Nse)-Pten KO mice, the authors mistook the prepulses at 4dBm 8dB and 16dB as the actual magnitude of the prespulses used by Kwon et al. In fact, these refer to prepulse of intensity at 4, 6 and 18 decibels units above background. The use of 70, 75 and 80dB prepulses here were presented against a background noise level of 68dB, and thus effectively be +2, +7 and +12 decibel units above background. Hence, it is incorrect to conclude (by comparison between the present study and Kwon et al.) that “there may only be changes in percent inhibition in Pten mutant mice when the prepulse in comparatively quiet”.  If anything, the +2 (or 70 dB) condition here was even lower than the weakest prepulse used by Kwon et al.

-  Thank you for pointing this out. The discussion has been amended with a more specific interpretation of Kwon et al’s (2006) findings. Furthermore, additional explanations have been made to explain any differences in results.

 

The authors evaluated the startle reactivity curve as a function of increasing pulse intensity – as a means to examine the “startle threshold”. Yet no attempt was made to measure individual startle threshold for comparison between genotypes. Otherwise, it is misleading to conclude that threshold did not differ when only the group’s average profile was presented.

- We believe that the repeated measures ANOVA with genotype as the between-subjects factor and stimulus intensity as the within subjects factor is sufficient to adequately, and thoroughly, assess the data.  Specific details further explaining our statistics have been added to the paper to help clarify any confusion.    
